# Perspectives on Peripheral Neuropathy as a Consequence of Metformin-Induced Vitamin B12 Deficiency in T2DM

**DOI:** 10.1155/2017/2452853

**Published:** 2017-08-27

**Authors:** Marwan A. Ahmed, George L. Muntingh, Paul Rheeder

**Affiliations:** ^1^Department of Pharmacology, Faculty of Health Sciences, University of Pretoria, Pretoria, South Africa; ^2^Department of Internal Medicine, Steve Biko Academic Hospital, University of Pretoria, Pretoria, South Africa

## Abstract

Peripheral neuropathy (PN) is a primary complication of type 2 diabetes mellitus (T2DM) and a direct manifestation of vitamin B12 deficiency. Examining the effects of metformin use on PN status became imperative following clinical studies that showed the vitamin B12-lowering effect of the medication. The complexity of the topic and the inconsistency of the results warrant consideration of topic-specific perspectives for better understanding of the available evidence and more appropriate design of future studies.

## 1. Introduction

T2DM is a metabolic disorder that is increasingly becoming a public health concern. The disease is associated with a variety of systemic macrovascular and microvascular complications. Diabetic peripheral neuropathy (DPN) is the most common complication, and it may eventually develop in up to 50% of patients [1]. DPN is associated with ulcerations, recurrent foot infections, Charcot's joints, foot or ankle fractures, amputations, and depression. As a result, DPN can cause physical limitations, increased utilization of health care, and diminished work productivity [2]. Gordois et al. estimated the annual expenditure on DPN in the United States to be between 4.6 and 13.7 billion dollars [3]. In addition, 27% of diabetes-related medical costs are attributable to DPN [3].

Both American and European guidelines recommend metformin as the first-line agent for the pharmacological management of T2DM. Accumulating evidence suggests that long-term use of metformin is associated with low vitamin B12 levels, and findings from both observational and interventional studies have confirmed this association [4–8]. Recent American Diabetes Association (ADA) guidelines recommend periodic testing of vitamin B12 in metformin-treated patients, especially in those with peripheral neuropathy [9].

Since vitamin B12 is essential for the remethylation of homocysteine to methionine, metformin-induced vitamin B12 deficiency could be associated with hyperhomocysteinemia, a condition with a questionable detrimental impact on macrovascular disease in T2DM patients [10]. The clinical presentation of vitamin B12 deficiency generally includes haematological and neurological manifestations. Neuropathy can be the only manifestation of the deficiency, without a haematologic presentation [11]. Over the last few decades, the clinical manifestations of vitamin B12 deficiency have shown notable trends towards neurological signs and symptoms [12]. PN resulting from vitamin B12 deficiency is clinically indistinguishable from DPN [13]. Such PN neuropathy can be asymptomatic [12] and could likely worsen DPN in diabetic patients. It is reasonable to assume that PN resulting from metformin-associated vitamin B12 deficiency has a significant risk of being misdiagnosed as DPN. Long-term metformin use can theoretically cause or worsen PN in diabetes patients, adding to its considerable burden.

The potential for metformin-induced vitamin B12 deficiency to cause or worsen DPN has recently been investigated by several studies with conflicting findings. This article provides new perspectives that may assist in interpreting the available evidence and give a deeper understanding of the topic for the design of future research.

## 2. The Current Evidence

The potential association between metformin use and vitamin B12 deficiency reported by interventional and observational studies has enabled researchers to question whether such an association can have clinical implications. Recently, the topic has attracted attention, and several studies were conducted to investigate the potential for metformin-induced vitamin B12 deficiency to cause, or worsen, PN in T2DM patients.

The insidious nature of neuropathy makes clinical trials and cohort studies impractical to investigate the possible relationship between metformin use and worsening of PN in T2DM patients [14]. Ethical, sample size, and study duration considerations oppose utilizing clinical trials to answer the question [14]. Despite their questionable validity and known limitations, cross-sectional and case-control studies seem to be the most convenient for studying the relationship. Thus, all of the available evidence comes from such studies. The results were conflicting and studies have shown differences in designs and settings (Table 1). Neuropathy was assessed by different tools with various degrees of subjectivity, and all studies had relatively small sample sizes (Table 1).

## 3. Antineuropathic and Neuroprotective Effects of Metformin

Animal studies have recently shown that metformin can exert neuroprotective and antineuropathic activities that are independent of its euglycemic effect. Metformin protects against numbness and neuropathic pain induced by chemotherapy in mice [21]. The sensory symptoms of pain, dysaesthesia, and paraesthesia characterize both diabetic PN and chemotherapy-induced PN, signifying the impact of these findings. Animal studies have also reported that metformin abolished pain resulting from the activation of sensory neurons [22] as well as resolved neuropathic allodynia [23], protected against ethanol-induced neuronal apoptosis [24], and enhanced neurogenesis [25]. It also suppressed cortical neuronal apoptosis [26] and exerted neuroprotective effects in Parkinson's disease [27]. Clinically, a retrospective chart review also reported the association between metformin use and a decrease in lumbar radiculopathy pain [28]. Antineuropathic effects of metformin may be mediated by 5′ adenosine monophosphate-activated protein kinase (AMPK) activation [29]. Impaired AMPK signaling was linked to PN in animals [30].

The glycemic control-independent neuroprotective effect of metformin may add a new perspective to PN due to metformin-induced vitamin B12 deficiency [14]. Given the above evidence, metformin can influence PN in two ways, excluding that related to glycemic control. It may exert a positive neuroprotective effect, or it may enhance PN by inducing vitamin B12 deficiency. The results showing no association between vitamin B12 and PN do not preclude the ability of metformin to exert negative effects on PN status by lowering vitamin B12 levels. The vitamin B12 deficiency-mediated effects may blunt the antineuropathic, neuroprotective, and euglycemic actions, which partly blunt the medication's additional beneficial actions in PN.

The theory may provide deeper insight into the contradicting results of studies investigating PN in metformin-treated T2DM patients. The inconsistency of results may be attributed to variation in the individual weights of metformin's direct impact on the neurons, its metabolic euglycemic effect, and its vitamin B12 deficiency-mediated neuropathic effect. It can be hypothesized that the findings in each study reflect the dominant action of metformin in that particular population. The sample and study characteristics, such as the mean age, duration of diabetes, metformin dose, and duration as well as race and comorbidities, may contribute to the dominance of certain axes and to the net impact of metformin on PN.

## 4. Study Design and Methodological Issues

Factors within clinical practice might have methodological impacts that deserve consideration when interpreting the evidence on PN resulting from metformin-associated vitamin B12 deficiency [14]. Both American and European guidelines recommend using metformin as a first-line medication for T2DM. Encountering T2DM patients who are not on metformin is uncommon. Metformin users and nonusers are anticipated to be inherently different in observational studies that compare the two groups. Obtaining similar study groups can become unattainable by having one group with the “abnormality” of not taking metformin. The highly probable and adherent imbalance between the two comparison groups makes it unclear whether metformin use has played the primary role in the outcome and worsening of PN. In other words, factors distributed differentially between the two groups can provide alternative explanations for the findings, thus threatening the study validity. A control group may discredit the validity in studies aiming at investigating the effect of metformin-induced vitamin B12 deficiency on PN. Even comparison groups can only be attained by randomization in controlled trials, and such study designs are impractical to answer this question.

Considering the antineuropathic and neuroprotective actions of metformin also gives us a different view regarding the designs of studies aiming at exploring the relationship between medication use and PN. In the metformin group, there is a possibility that the medication's antineuropathic and neuroprotective effects predominate or dilute its vitamin B12 deficiency-mediated neuropathic impact. Thus, comparing PN in the metformin and nonmetformin groups could produce distorted conclusions that do not reflect the real contribution of metformin-induced vitamin B12 deficiency to PN. This perspective adds strength to the studies that compared vitamin B12-normal and -deficient patients using metformin and discredits the classical designs comparing metformin users and nonusers.

## 5. An Emerging Theory on Metformin-Induced Vitamin B12 Deficiency

Vitamin B12 is involved in both methylmalonyl-CoA mutase and methionine synthase intracellular pathways. Vitamin B12 deficiency interferes with the two pathways and causes increased levels of methylmalonic acid and homocysteine, which are also considered as biochemical indicators of cellular (metabolic) vitamin B12 deficiency [31]. The increase in homocysteine concentration can also be a result of folic acid deficiency [31].

The theory that metformin only lowers the circulating vitamin B12 without affecting the intracellular vitamin levels was recently introduced [32]. This theory is based on the fact that low vitamin B12 status should result in increased levels of the biochemical indicators of metabolic deficiency. Clinical studies, which reported nonelevated concentrations of methylmalonic acid and homocysteine among metformin-treated patients, constitute the framework of the theory. Reinstatler et al. found that despite their significantly lower levels of vitamin B12, metformin users had lower concentrations of homocysteine when compared to nonusers [33]. As the study was conducted after the commencement of the folic acid fortification in the United States, homocysteine levels carried more diagnostic specificity in assessing vitamin B12 status. The theory also relies on the controlled trial of de Jager et al. which found no significant increase in homocysteine concentrations despite low vitamin B12 levels in the metformin group when studied over a period of 4.3 years [4]. Greibe et al. reported that a six-month treatment with metformin resulted in lower serum vitamin B12 without an impact on MMA in women with polycystic ovary syndrome [34]. The authors took a step further and measured vitamin B12 bound to haptocorrin, a transporter that binds 70% to 80% of the circulating vitamin forming a metabolically inert complex. The results showed a reduction in haptocorrin-bound vitamin levels in the metformin group. They concluded that metformin-induced low vitamin B12 levels resulted from the decrease in B12-haptocorrin fraction and did not reflect a true metabolic deficiency. An animal study has also reported increased liver accumulation and decreased circulating concentrations of vitamin B12 in rats following subcutaneous administration of metformin [35]. The authors proposed that metformin enhanced redistribution of vitamin B12 rather than affecting its cellular status.

Adopting the above theory would mean that metformin does not cause or worsen PN in diabetic patients. However, substantial evidence contradicts the theory. The difference in homocysteine levels in the metformin and nonmetformin groups was not statistically significant in the study of Reinstatler et al. [33]. The trial of de Jager et al. indeed reported that metformin caused a slight increase of 5% in homocysteine levels. The *p* value was 0.09, which may indicate a borderline significant trend. The trial's authors attributed the nonsignificance to the relatively low numbers of patients with vitamin B12 deficiency, and they expected the homocysteine levels to show further increases with a longer treatment duration [4]. Moreover, the six-month period of metformin use in a study by Greibe et al. did not seem to be sufficient to deplete vitamin B12 stores and consequently result in elevated MMA or homocysteine levels. Most importantly, the association between metformin use and the increased levels of cellular vitamin B12 biomarkers was reported by many studies [15, 20, 36–38]. Moving to clinical practice, the aim of clinical research, accepting the theory that metformin only reduces circulating B12 and not its intracellular levels, may take us to another level and raises a crucial question on the validity of the current vitamin B12 serum test. Adopting this theory dictates a revolutionary approach in testing B12 in clinical settings. The theory, however, raises the importance of showing the potential of metformin to cause cellular vitamin B12 deficiency as an achievable initial goal of future research.

## 6. Other Perspectives

The clinical trial of de Jager et al. has shown that long-term metformin treatment gradually lowered the levels of vitamin B12 [4]. Thus, the possible development of PN due to metformin-induced vitamin B12 deficiency is anticipated to be insidious and progressive. Long periods of treatment with metformin in studies with sufficiently large sample sizes may be required to reveal a detectable and statistically significant PN.

Vitamin B12 levels may differ in various ethnic groups. Several studies reported higher concentrations of vitamin B12 in black individuals when compared to white individuals [39–41]. This is attributed to higher levels of vitamin B12-binding proteins in the black populations [42]. We have recently reported higher concentrations of the vitamin among black South African T2DM patients on metformin [14]. To our knowledge, the impact of ethnicity on the cellular status of the vitamin is not yet investigated. Currently, utilized cutoff points and definitions of vitamin B12 deficiency do not consider the possible effects of ethnicity. Ethnicity should be taken into consideration as a contributing factor when studying PN as a clinical consequence of metformin-induced vitamin B12 deficiency.

## 7. Conclusion

The conflicting results of the available evidence reflect the complexity of linking metformin-induced vitamin B12 deficiency and PN in T2DM patients, where the disease state also induces neuropathies. Different perspectives can be considered in interpreting and designing studies attempting to explore the triangle of metformin, vitamin B12, and PN in T2DM. The glycemic control-independent neuroprotective and antineuropathic effects of metformin recently reported in animal studies may explain the contradicting nature of the obtained results and enhance understanding of the topic. Obtaining similar study groups is probably unachievable in observational studies that compare metformin users and nonusers, which blunts their validity and raises questions about most of the available evidence.

Future research should probably first focus on confirming metformin's potential to cause vitamin B12 metabolic deficiency. Studies with sufficiently large sample sizes and proper designs that utilize more objective, and conventional, PN-assessing tools are required to investigate the relationship between metformin use and PN in T2DM patients.

## Figures and Tables

**Table 1:** Settings, designs, and results of studies that investigated the impact of metformin-induced low vitamin B12 on peripheral neuropathy in T2DM patients.

Study	Setting	Design	Results
Wile and Toth [15]	Neuromuscular clinic at a university hospital, Canada	Case-control study. Cases were T2DM patients on metformin with primary diagnosis of PN (59 participants). Controls were T2DM patients not taking metformin with primary diagnosis of PN (63 participants).	The metformin group had more severe PN (assessed by TCSS and NIS). Electrophysiological markers showed no significant difference between the two groups. Cumulative metformin dose showed a significant positive correlation with TCSS scores (rho = 0.80) and NIS scores (rho = 0.79).
Singh et al. [16]	Internal medicine clinic in a tertiary hospital, India	Cross-sectional study. Randomly selected T2DM patients were divided into metformin users (84 participants) and nonusers (52 participants).	The metformin group had more severe PN (assessed by TCSS). Cumulative metformin dose revealed a significant positive correlation with TCSS (rho = 0.53).
de Groot-Kamphuis et al. [17]	Secondary care outpatient diabetes clinic, the Netherlands	Cross-sectional study. Randomly selected T2DM patients were divided into metformin users (164 participants) and nonusers (134 participants).	Prevalence of neuropathy (obtained from records) was significantly lower in the metformin group.
Chen et al. [13]	Diabetes clinic of a tertiary hospital, UK	Cross-sectional study. Randomly selected T2DM patients were divided into metformin users (152 participants) and nonusers (50 participants).	All PN-assessing tools (monofilament, neurothesiometry, NTSS-6, and s-LANSS) showed no significant differences between the two groups.
Biemans et al. [18]	Four primary care centers, the Netherlands	Cross-sectional study. Metformin-treated T2DM patients were divided into the vitamin B12-deficient (126 participants) and normal (322 participants) groups.	There were no significant differences in PN (assessed by MNSI and extracted from records) between the two groups.
Russo et al. [19]	Diabetes clinic of a university hospital, Italy	Cross-sectional study. T2DM patients were divided into metformin users (124 participants) and nonusers (139 participants).	There was no significant difference in prevalence of PN between the two groups. PN was suspected based on abnormalities of certain evaluations and confirmed by NCVs.
Roy et al. [20]	Tertiary Hospital, India	Cross-sectional study. T2DM patients were divided into (1) the metformin group (35 participants), (2) the metformin + other antihyperglycemic group (20 participants), and (3) the nonmetformin group (35participants).	Neuropathy (assessed by NCVs) did not differ significantly between the groups.
Ahmed et al. [14]	Diabetes clinics of two tertiary hospitals, South Africa	Cross-sectional study. Metformin-treatedT2DM patients were divided into the vitamin B12-deficient (34 participants) and normal (87 participants) groups.	There was no difference in the presence of PN (assessed by NTSS-6) between the two groups. Levels of vitamin B12 and NTSS-6 scores were not correlated.

MNSI: Michigan Neuropathy Screening Instrument; NCVs: nerve conduction velocities; NIS: Neuropathy Impairment Score; NTSS-6: Neuropathy Total Symptom Score-6; PN: peripheral neuropathy; rho: Spearman's rank correlation coefficient; s-LANSS: Self-administered Leeds Assessment of Neuropathic Symptoms and Signs; TCSS: Toronto Clinical Scoring System.
